# Nitric Oxide Donor NOC-18-Induced Changes of Mitochondrial Phosphoproteome in Rat Cardiac Ischemia Model

**DOI:** 10.3390/medicina55100631

**Published:** 2019-09-24

**Authors:** Danielius Umbrasas, Ramūnas Jokubka, Algirdas Kaupinis, Mindaugas Valius, Odeta Arandarčikaitė, Vilmantė Borutaitė

**Affiliations:** 1 Neuroscience Institute, Lithuanian University of Health Sciences, LT-44307 Kaunas, Lithuania; danielius.umbrasas@lsmuni.lt (D.U.);; 2 Proteomics Center, Institute of Biochemistry Vilnius University Life Sciences Center, Vilnius University, LT-10257 Vilnius, Lithuania

**Keywords:** heart ischemia, nitric oxide, mitochondrial permeability transition pore, phosphoproteome, preconditioning, ATP synthase

## Abstract

*Background and objective*: Nitric oxide (NO) is known to exert cardioprotective effects against heart ischemic damage and may be involved in ischemic pre- and postconditioning. NO-triggered cardioprotective mechanisms are not well understood but may involve regulation of mitochondrial permeability transition pore (mPTP). In this study, we aimed to identify differentially phosphorylated mitochondrial proteins possibly involved in the NO/protein kinase G (PKG)/mPTP signaling pathway that can increase the resistance of cardiomyocytes to ischemic damage. *Materials and methods*: Isolated hearts from Wistar rats were perfused with NO donor NOC-18 prior to induction of stop–flow ischemia. To quantify and characterize the phosphoproteins, mitochondrial proteins were resolved and analyzed by two-dimensional gel electrophoresis followed by Pro-Q Diamond phosphoprotein gel staining, excision, trypsin digestions, and mass spectrometry. Quantitative proteomic analysis coupled with liquid chromatography–tandem mass spectrometry was also performed. *Results*: Mitochondrial protein phosphorylation patterns in NOC-18-pretreated ischemic hearts versus ischemic hearts were compared. Pretreatment of hearts with NOC-18 caused changes in mitochondrial phosphoproteome after ischemia which involved modifications of 10 mitochondrial membrane-bound and 10 matrix proteins. Among them, α-subunit of ATP synthase and adenine nucleotide (ADP/ATP) translocase 1, both of which are considered as potential structural components of mPTP, were identified. We also found that treatment of isolated non-ischemic mitochondria with recombinant PKG did not cause the same protein phosphorylation as pretreatment of hearts with NOC-18. *Conclusions*: Our study suggests that pretreatment of hearts with NOC-18 causes changes in mitochondrial phosphoproteome after ischemia which involves modifications of certain proteins thought to be involved in the regulation of mPTP opening and intracellular redox state. These proteins may be potential targets for pharmacological preconditioning of the heart.

## 1. Introduction

Ischemic heart pathologies including acute myocardial infarction which can result in heart failure are the leading cause of mortality and disability worldwide. In clinical settings, timely myocardial reperfusion after the ischemic period is the main therapeutic strategy for reducing infarct size and prevention of the development of heart failure, however, this strategy is not efficient enough for all patients. Therefore, new strategies and pharmacological means are needed for further improvement of treatments of heart ischemic pathologies. To achieve that, an understanding of cellular and molecular mechanisms underlying the ischemic heart damage as well as cardioprotective processes is urgently needed.

It is generally accepted that mitochondria play a crucial role in the pathogenesis of multiple cardiac diseases including ischemic heart disease and myocardial infarction, mainly due to the imbalance of cellular bioenergetics (loss of ATP synthesis and increase of ATP hydrolysis), impairment in ionic homeostasis (intracellular Ca^2+^ in particular), formation of reactive oxygen species (ROS) and release of proapoptotic proteins—all being considered as key factors in the process of irreversible heart damage [1,2,3]. Mitochondrial dysfunction impairs their communication with other cellular organelles leading to alteration in energy homeostasis that affects whole cellular metabolism and cause cell death. However, relevant questions concerning detailed molecular mechanisms and causal relationships still remain unsolved.

Mitochondrial permeability transition pore (mPTP) opening has been suggested as one of the main mechanisms in ischemic heart damage [3], and regulation of mPTP in both ischemic pre- and postconditioning has been proposed as the main endogenous mechanism in cardioprotection [2,3,4,5]. In pathological conditions, mitochondrial Ca^2+^ uptake in the cytoplasm results in opening of mPTP in the mitochondrial inner membrane leading to osmotic swelling of organelles followed by cellular degeneration and death [1,3]. Thus, mPTP plays a central role in maintaining the balance between cell survival and death. Mitochondrial PTP opening is regulated by multiple molecular effectors, such as Ca^2+^, oxidizing agents and ROS, including NO [6]. It is well described that NO is a ubiquitous cellular messenger that is synthesized in biological systems by NO synthases (NOS) [7] and is involved in mitochondrial biogenesis, regulation of energy metabolism and cell cycling [8]. NO is also an important vasodilator and is involved in heart pharmacological or ischemic preconditioning [8]. Previously we have shown that pretreatment of hearts with NO donor NOC-18 reduced ischemia- or ischemia/reperfusion-induced injury to cardiac mitochondria and prevented apoptotic cell death [9]. These effects were shown to be mediated by NO-activated protein kinase G (PKG) and protein kinase C (PKC) possibly acting on and preventing opening of mPTP [9]. However, which particular components of mPTP could be modified by NO/PKG/PKC has not been determined yet. It is an important question, as understanding how mPTP can be regulated may help to develop new strategies and therapeutic means for treatment of ischemic heart pathologies. In order to better understand NO-induced protective mechanisms operating in cardiomyocytes under ischemia we quantitatively analyzed changes in mitochondrial phosphoproteome after 30 min of ischemia, induced by pretreatment of the hearts with NO donor NOC-18, and sought to determine whether potential structural components of mPTP are affected by pretreatment of hearts with NOC-18.

## 2. Materials and Methods

### 2.1. Materials

The Pro-Q Diamond Phosphoprotein Enrichment kit/reagents and NuPAGE precast 4%–12% Bis–Tris polyacrylamide gels were purchased from Life Technologies. Bradford Protein concentration assay kit and TPCK Trypsin 20233 for protein digestion were obtained from Thermo Fisher. Protease and phosphatase (for serine, threonine, and tyrosine) inhibitors and all other reagents were of analytical grade and quality and were obtained from Sigma (if not stated otherwise).

### 2.2. Experimental Animals and Induction of Heart Ischemia

Experimental procedures involving animals were undertaken in accordance with the EU Directive 2010/63/EU for animal experiments and the Republic of Lithuania law on the care, keeping, and use of experimental animals (Approved by Lithuanian State Food and Veterinary Service, ethical approval No. B6(1.9)-855). Experiments were performed on hearts isolated from 60 to 90 days old male Wistar rats randomly distributed into two groups: ischemic and NOC-18-pretreated. Animals were bred and kept under controlled environmental conditions with a 12 h light/12 h dark cycle, at a constant temperature of 22 ± 1 °C. They were housed in plastic cages (three animals per cage) with water and food ad libitum in the breeding and housing facilities at the Center of Biological Research of Lithuanian University of Health Sciences.

Rats were killed by increasing the concentration of CO_2_ in the air followed by cervical dislocation. Hearts were rapidly excised and perfused on a Langendorff perfusion system with Krebs–Henseleit solution (11 mM glucose, 118 mM NaCl, 25 mM NaHCO_3_, 4.8 mM KCl, 1.2 mM KH_2_PO_4_, 1.2 mM CaCl_2_, 1.7 mM MgSO_4_, and 0.7 mM Na pyruvate, saturated with 95% O_2_ and 5% CO_2_, pH 7.4 at 37 °C) at a pressure of 80 cm H_2_O. After a 15 min equilibration period, hearts were perfused with 1 µM NOC-18 for 4 min followed by stop–flow 30 min global ischemia as described previously [9].

### 2.3. Mitochondrial Preparation

Immediately after ischemia mitochondria were isolated by homogenizing heart tissues with a glass–Teflon homogenizer in the medium (10 mL/g of tissue) containing 160 mM KCl, 10 mM NaCl, 20 mM TrisHCl, 2 mM EGTA, pH 7.7 followed by differential centrifugation (5 min × 1000 *g* and 10 min × 6800 *g*). All procedures with mitochondria were performed at 4 °C temperature. The mitochondrial pellet was resuspended in the isolation medium and total mitochondrial protein was determined by the modified Biuret method [10]. For mitochondrial proteome experiments, mitochondrial proteins were extracted with a solubilization buffer containing 7 M urea, 2 M thiourea, 40 mM DTT, 4% CHAPS. Prior to the isolation of proteins, protease and serine/threonine phosphatase inhibitors were added into the protein extraction buffer. The ratio of buffer to mitochondria pellet was approx. 3:1 (v/v). After mitochondria were lysed, the samples were sonicated (80 W, 1 s ultrasonic at a time, 10 times). The mitochondrial protein content in the samples were quantified by the Bradford method [10] and stored at −80 °C until further applications.

### 2.4. Mitochondrial Incubation with PKG

In experiments testing the effects of PKG, mitochondria were isolated from normal (non-ischemic) rat hearts. Isolated mitochondria were suspended at 15 mg/mL concentration in the isolation buffer containing 160 mM KCl, 10 mM NaCl, 20 mM Tris HCl, 2 mM EGTA, pH 7.7 at 4 °C. Then two volumes of water were added to one volume of mitochondrial suspension and mitochondria were preincubated in these hypotonic conditions for 1–2 min. Then 500 μM pyruvate plus 500 μM malate, 100 μM cyclic guanosine monophosphate (cGMP), 100 μM ATP, and 40 U/mL PKGIα (recombinant; from Merck Millipore) were added and incubated for 15 min at room temperature. Control mitochondria were incubated in the same conditions but without PKG. After the incubation, mitochondria were collected by centrifugation (6800 × *g* for 10 min) and followed by the mitochondria lysis and protein extraction procedure.

### 2.5. Phosphoprotein Enrichment

Solubilized mitochondrial lysates were diluted and loaded onto a column pre-equilibrated with Pro-Q Diamond phosphoprotein-binding resin. The column was washed, and phosphoproteins were eluted with elution buffer then concentrated by centrifugation in Vivaspin filtration concentrators with a 10 kDa cutoff polyethersulfone membrane. Concentrated mitochondrial phosphoproteins were stored at −80 °C until further used. Two-dimensional gel electrophoresis was performed on the Novex NuPage system. In brief, samples were prepared, and first-dimension runs were performed on 7 cm precast strips (pH range 3–10) using the ZOOM IPGRunner isoelectric focusing system according to the manufacturer’s instructions. After isoelectric focusing, strips were run using Novex NuPage 4%–12% Bis–Tris ZOOM precast gels at 200 V for 50 min. Phosphoproteins on the gels were stained with Pro-Q Diamond and total proteins were stained with SYPRO Ruby stains as indicated in manufacturer’s recommendations. Stained gels were scanned on a Bio-Rad Molecular Imager FX, and comparative analysis of mitochondrial protein profile between ischemic and ischemic/NOC-18 groups was performed with analytical Prodigy 2D software.

### 2.6. In-Gel Protein Digestion for Mass Spectrometry Analysis

In-gel trypsin digestion was performed according to a protocol described by Hellman et al. [11] with minor modifications. Briefly, gel slices were destained with 200 µL of 50 mM ammonium bicarbonate in 50% CH_3_CN, vacuum dried, rehydrated in 50 µL (20 µg/mL) of trypsin TPCK Trypsin 20233 containing 25 mM NH_4_HCO_3_ and incubated overnight at 37 °C. The peptides were extracted from the gel using 100 µL of CH_3_CN for 30 min. Next, gel pieces were washed with 100 µL of 1% formic acid for 10 min. The extraction procedure was finished by adding 100 µL of CH_3_CN. The peptides from all extractions were combined, acidified, concentrated by vacuum drying, resuspended in 40 µL 0.1% formic acid, and then used for mass spectrometry (MS) analysis.

### 2.7. Protein Digestion and Sample Preparation for LC–MS/MS Analysis

For mass spectrometry analysis, protein digestion was performed by using the filter-aided sample preparation (FASP) method [12]. Briefly, eluted mitochondrial phosphoproteins were diluted with 8 M urea acidified buffer (UA) containing 100 mM dithiothreitol in 150 mM Tris-HCl at pH 8.0, then alkylated using 50 mM iodoacetamide in UA buffer. Afterward, two washing steps with 50 mM NH_4_HCO_3_ were performed on samples, followed by overnight protein digestion with TPCK Trypsin 20233. Then peptides were recovered by centrifugation and washed twice with 50% CH_3_CN. Peptides prior to liquid chromatography – mass spectrometry (LC–MS) analysis were combined, acidified, lyophilized, redissolved in 0.1% formic acid, and analyzed as described previously [13].

### 2.8. Liquid Chromatography and Mass Spectrometry

Liquid chromatography (LC) separation of trypsin-cleaved peptides was performed with nanoAcquity UPLC system (Waters Corporation, Wilmslow, UK). Peptides were loaded on a reversed-phase trap column PST C18, 100 Å, 5 µm, 180 µm × 20 mm (Waters Corporation, Wilmslow, UK) at a flow rate of 15 µL/min using loading buffer of 0.1% formic acid and subsequently separated on HSS-T3 C18 1.8 µm, 75 µm × 250 mm analytical column (Waters Corporation, Wilmslow, UK) in 30 min linear gradient (A: 0.1% formic acid, B: 100% CH_3_CN, and 0.1% formic acid) for in-gel protein trypsin digested samples or 60 min for FASP material at a flow rate of 300 nL per min. The analytical column temperature was kept at 40 °C. The nano-LC was coupled online through a nano-ESI 7 cm length, 10 mm tip emitter (New Objective, Woburn, MA, USA) with HDMS Synapt G2 mass spectrometer (Waters Corporation, Wilmslow, UK). Data were acquired using MassLynx version 4.1 software (Waters Corporation, Wilmslow, UK) in positive ion mode. LC–MS data were collected using data independent acquisition (DIA) mode MSE (for in-gel digested proteins) or MSE in combination with online ion mobility separations (for FASP material). The trap collision energy of mass spectrometer was ramped from 18 to 40 eV for high-energy scans in MSE mode. The trap and transfer collision energy for high-energy scans in HDMS mode was ramped from 4 to 5 eV and from 27 to 50 eV. For both analyses, the mass range was set to 50–2000 Da with a scan time set to 0.9 s. A reference compound [Glu1]-Fibrinopeptide B (Waters Corporation, Wilmslow, UK) was infused continuously (500 fmol/µL at flow rate 500 nL per min) and scanned every 30 s for online mass spectrometer calibration purpose.

### 2.9. Data Processing, Searching, and Analysis

Raw data files were processed and searched using ProteinLynx Global SERVER (PLGS) version 2.5.2 (Waters Corporation, Wilmslow, UK). The following parameters were used to generate peak lists: (i) Minimum intensity for precursors was set to 100 counts, (ii) minimum intensity for fragment ions was set to 30 counts, (iii) intensity was set to 500 counts. Processed data were analyzed using trypsin as the cleavage protease, one missed cleavage was allowed, and fixed modification was set to carbamidomethylation of cysteines, variable modification was set to oxidation of methionine. Minimum identification criteria included two fragment ions per peptide, five fragment ions per protein and minimum of two peptides per protein. The false discovery rate (FDR) for peptide and protein identification was determined based on the search of a reversed database, which was generated automatically using PLGS when the global false discovery rate was set to 4%.

### 2.10. Statistical Data Analysis

Before comparative analysis data filtering was performed according to strict criteria: data were removed from the analysis if the protein was quantifiable in only one of three replicates in one experimental group. Then data were normalized and analyzed with Bioconductor packages in R 3.6.0 software to perform comparative statistics between ischemia and ischemia/NOC-18 groups. To avoid false-positives, a 1.5-fold change cutoff value was set in the comparative analysis. To evaluate segregation among experiments, we performed principal component analysis (PCA) for dimensionality of matrix consisting of the objects from three biological experiments of ischemic hearts and two biological experiments with hearts perfused with NOC-18 before ischemia. Each experiment was performed with three technical replicates. The PCA results showed that experiments and each technical replicate segregate to their corresponding groups that represent the model reproducibility. Proteins were considered differentially phosphorylated if their levels were at least 1.5-fold different between groups and Student’s *t*-test *p*-values were <0.05.

## 3. Results

### 3.1. Mitochondrial Phosphoproteins in Response to NOC-18 versus Ischemia

**Figure 1:**
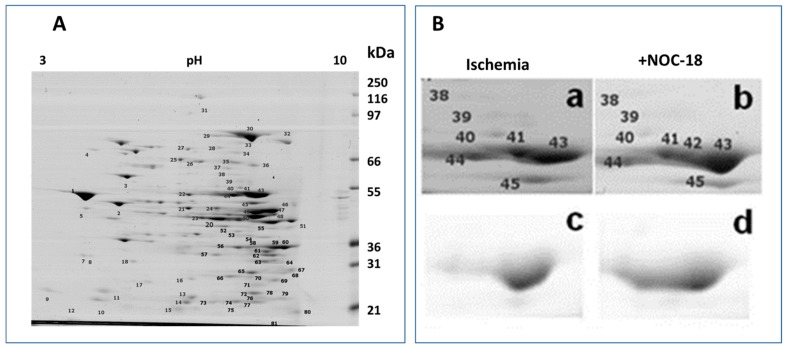
Mitochondrial protein profiles on 2-DE gels. (**A**) A representative 2-DE gel profile of the whole mitochondrial proteome stained with SYPRO^®^ Ruby Protein Gel Stain. Numbers on the gel indicate 80 protein spots which exhibited different phosphorylation levels between ischemic and NOC-18-treated groups. (**B**) Regions of the gels containing spots 38–45: The panels (**a**) and (**b**) represent total protein stained with SYPRO^®^ Ruby Protein Gel Stain; and panels (**c**) and (**d**) represent phosphoprotein staining with Pro-Q^®^ Diamond Phosphoprotein Gel Stain. Spots were excised from gels for protein identification by matrix assisted laser desorption/ionisation - time of flight/time of flight high resolution tandem mass spectrometry (MALDI–TOF/TOF MS/MS).

To determine which mitochondrial proteins are phosphorylated after ischemia and pretreatment with NO donor NOC-18, 2-DE gels were stained with Pro-Q Diamond for phosphoproteins and with SYPRO Ruby for total protein. Using this approach about 80 protein spots, which exhibited different phosphorylation levels (staining of gels with Pro-Q Diamond) between ischemic and NOC-18-treated groups, were determined. Further densitometric analysis of these spots revealed that only six protein spots from two experiments showed higher than 1.5-fold differences in relative spot volume between ischemic and ischemic plus NOC-18 mitochondrial samples. These spots were excised from the gels and analyzed by mass spectrometry. As presented in Figure 1, the protein spot belonging to ATP synthase α subunit (ATP5A1, spot 42) was identified with high reliability in both gels (NOC-18-treated and not treated heart mitochondrial samples). Three other proteins in spots were identified with lower reliability or were identified only in one gel: NADH dehydrogenase (ubiquinone) flavoprotein 2 (NDUFV2, spot 13), ATP synthase d subunit (ATP5H, spot 15), and propionyl-coenzyme A carboxylase α chain (PCCA, spot 27) in Figure 1A. As shown in Figure 1B, relative spot volumes of ATP synthase α subunit was increased after NOC-18 treatment of the hearts (Figure 1B(c,d), compare spot 42). However, complete statistical analysis performed on data from all five heart mitochondrial preparations did not show statistically significant difference in relative spot volumes of the identified proteins in ischemic and ischemia plus NOC-18 groups.

Therefore, we decided to perform global proteomics analysis of phosphoprotein-enriched mitochondrial fractions obtained from ischemic and ischemia plus NOC-18 hearts. There were 171 mitochondrial proteins in the phosphoprotein-enriched fraction identified and quantified (data not shown). The identified differentially phosphorylated proteins with at least 1.5-fold changes were categorized based on subcellular location using UniProt database (http://www.uniprot.org) and are presented in Table 1. Statistical analysis revealed that the phosphorylation level of 20 quantified proteins significantly differed: nine membrane-bound proteins (MPC1, ACADVL, SLC25A11, SLC25A4, ATP5A1, HADHB, ETFD, COX4I1, UQCRFS1, see Table 1) and 10 mitochondrial matrix proteins (PDHA1, ACADS, ACADVL, IVD, SUCLG1, LONP1, COQ3, CS, ACAA2, and MDH2, see Table 1) were significantly upregulated and one membrane-bound protein, NADH dehydrogenase ubiquinone flavoprotein 2 (NDUFV2) was downregulated in the pretreatment group with NOC-18 versus ischemia.

### 3.2. Mitochondrial Proteins after Incubation with PKG

**Figure 2:**
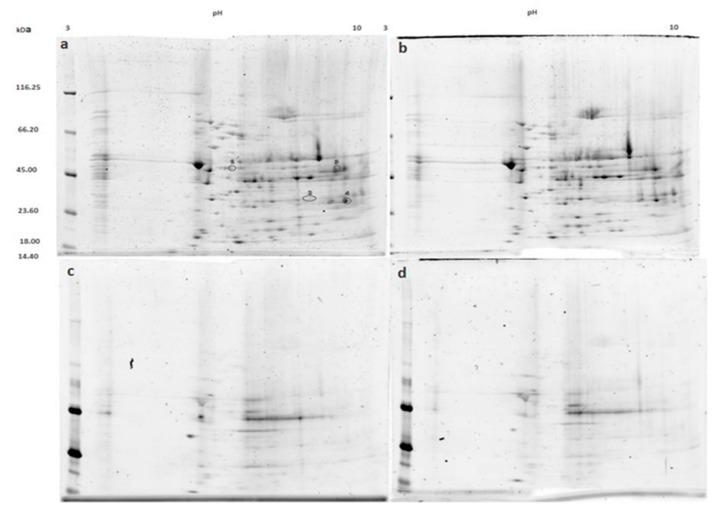
2-DE gels acquired from control and protein kinase G (PKG)-treated mitochondria. Gels a and b represent the whole mitochondrial proteome ((**a**) being the control and (**b**) PKG-treated). Gels c and d are stained with Pro-Q^®^ Diamond phosphoprotein stain and they represent phosphoprotein profiles of control (panel (**c**)) and PKG-treated (panel (**d**)) mitochondria. Densitometric analysis of the gels showed four protein spots (marked in panel (**a**)) that were significantly different between groups in the total protein gels, but no statistically significant differences were found in phosphoprotein profiles.

**Figure 3:**
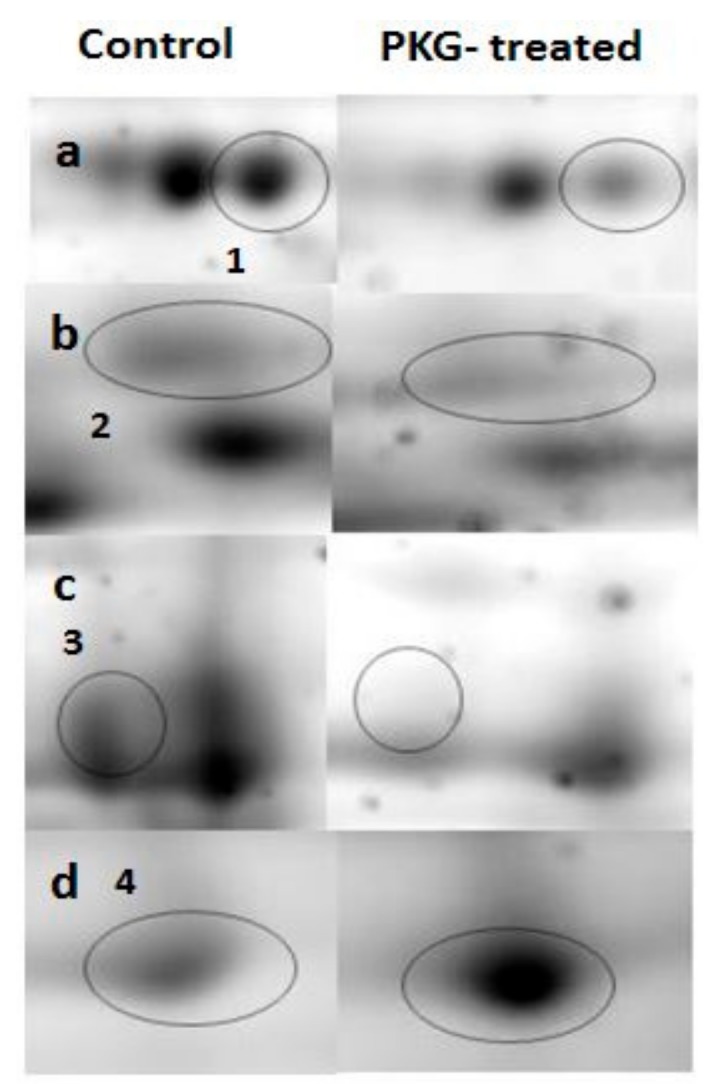
2-DE gel regions containing PKG-affected proteins. Row (**a**) shows 1.9-fold decrease in 2-oxoglutarate dehydrogenase complex (DLST) after incubation with PKG; row (**b**) 1.4-fold decrease in malate dehydrogenase (MDH); row (**c**) shows 1.8-fold decrease in a spot containing NADP-dependent isocitrate dehydrogenase (IDH2), ATP synthase subunit alpha and cytochrome b-c1 complex subunit 2 (UQCRC2); and row (**d**) represents 1.5-fold increase in a spot that contains VDAC proteins 1 and 3, Decr1 and Bdh1.

We treated normal mitochondria with PKG to investigate whether direct PKG action would result in similar changes in mitochondrial phosphoproteome as was found after pre-perfusion of hearts with NOC-18. Proteins from PKG-treated mitochondria and resolved on 2-DE gels showed no differences in the phosphoprotein profile compared to control (Figure 2c,d). However, some changes in the total mitochondrial proteome were found (Figure 3a,b). Spots marked 1, 2, and 3 have a lower density in the gels of PKG-treated mitochondria, while spot 4 shows a 1.5-fold increase in density after incubation with PKG. Spot 1 was identified as dihydrolipoyl-lysine-residue succinyltransferase component of 2-oxoglutarate dehydrogenase complex (DLST); spot 2—as malate dehydrogenase (MDH); spot 3 was identified to be NADP-dependent isocitrate dehydrogenase (IDH2), ATP synthase α subunit, and cytochrome b-c1 complex subunit 2 (UQCRC2). Spot 4 contains voltage-dependent anion channel (VDAC) proteins 1 and 3; 2,4-dienoyl-CoA reductase (DECR1), and D-beta-hydroxybutyrate dehydrogenase (BDH1).

## 4. Discussion

The main novel finding of our study was that we identified ten mitochondrial membrane-bound proteins and ten matrix proteins, phosphorylation levels of which were changed after the ischemic period due to pretreatment with NO donor NOC-18. Importantly, among identified significantly phosphorylated membrane-bound proteins, there were α-subunit of ATP synthase and ADP/ATP translocase 1. Both these enzyme complexes are considered as potential structural components of mitochondrial permeability transition pore [14]. We also found changes in phosphorylation of the components of mitochondrial Complex I (NADH dehydrogenase ubiquinone flavoprotein 2) and Complex III (electron transfer flavoprotein ubiquinone oxidoreductase mitochondrial) involvement of which in mPTP is less clear though there are recent data on regulation of mPTP by Complex I activity and by the redox state of coenzyme Q [15,16]. It is unclear, however, whether phosphorylation of these proteins, found in our study, affects their activities and/or their role in mPTP regulation and requires further investigations. Interestingly, the level of phosphorylation of Complex I component was found to be reduced, suggesting that the effect of NO may be possibly mediated by phosphatases. Complex I and Complex III are also involved in ROS production, therefore, NO-induced covalent modifications of their subunits may affect the cellular redox state during ischemia/reperfusion and may have important implications in myocardial damage or signaling in cardioprotection. The role of other phosphorylated membrane-bound and matrix enzymes in cardioprotection at the moment is unclear and not investigated.

The importance of NO/cGMP/PKG phosphorylation signaling pathway in cardioprotection is well recognized, however the precise mechanism and molecular targets of the signaling system are still widely negotiable. We and other investigators have shown importance of NO in promoting cell survival of cardiac and other tissues after ischemia/reperfusion [8,9,17]. In this study, in hearts pretreated with NO donor NOC-18 before ischemia, we observed changes in phosphorylation level of mitochondrial membrane-bound and matrix proteins, however, the protein phosphorylation pattern was not replicated by treatment of mitochondria with isolated PKG suggesting that mitochondrial proteins involved in fatty acid cycle and oxidative phosphorylation complexes might be modified indirectly through other NO-mediated pathways, e.g., involving cytosolic PKC [18] or ROS signaling. It has been reported that NO exerts some of its beneficial effects independently of PKG [8]. Interestingly, treatment of mitochondria with PKG caused changes in ATP synthase α-subunit content in the spot on the gel though this modification was not caused by phosphorylation of the protein since it was only observed in gels stained for total protein (not phosphoprotein specific). Such change in protein level in the protein spot on gel may be related to other possible covalent modifications of the proteins, e.g., oxidation or nitrosylation of some functional groups, causing shift of the protein on the 2-DE gel.

Incubation with PKG showed some changes in the whole mitochondrial proteome, mainly reflecting the mitochondrial redox state. It is suggested that the NO/cGMP/PKG signaling pathway involves opening of the mitoK_ATP_ channel which induces an increase in mitochondrial ROS generation [19,20]. In our experiments, PKG treatment resulted in decrease of spots densities of three proteins that are involved in mitochondrial redox regulation: DLST, IDH2, and MDH. 2-oxoglutarate dehydrogenase complex is an important mitochondrial redox sensor which can undergo oxidative modifications, particularly in its E2 subunit (DLST) [21]. IDH2 is a mitochondrial NADP-dependent enzyme which provides NADPH needed to regenerate the mitochondrial glutathione pool [22]. IDH2 is also shown to undergo acetylation/deacetylation in certain conditions, for example nutrient deprivation and caloric restriction [23]. MDH is an enzyme of the Krebs cycle which has also been shown to be sensitive to cellular redox state as well as undergo acetylation [24,25]. There was one protein spot on the 2-DE gel in which density was increased after PKG treatment. Four proteins were identified in the spot: VDAC1, VDAC3, decr1, and bdh1. VDAC proteins are implicated in the formation of mPTP [26] and modification of these proteins can be a regulatory mechanism for mPTP opening. As discussed above, the increase in this spot density cannot be attributed to phosphorylation because densitometric analysis of phosphoprotein gels revealed no differences between control and PKG-treated groups. The observed differences can be results of other types of covalent modifications, though we do not rule out the possibility that our methods for detection of protein phosphorylation were not sensitive enough to detect subtle changes in phosphorylation of the proteins.

In preclinical studies, mPTP opening is considered as a critical determinant of cell death during acute heart ischemia. However, in clinical studies cyclosporin A, an inhibitor of cyclophilin D—a component of mPTP—failed to exhibit beneficial effects [27]. This may be due to the narrow time-window for the application of this drug and therapeutic concentrations of the drug used in clinical studies. However, it is possible that new therapeutic means targeting other components of mPTP might be more effective against ischemic heart damage. Data of our study point to modifications of ATP synthase subunits and other mitochondrial proteins whose roles in cardioprotection are worth investigating further.

## 5. Conclusions

In summary, our study suggests that pretreatment of hearts with NO donor NOC-18 causes changes in mitochondrial phosphoproteome after ischemia which involves modifications of certain proteins thought to be involved in the regulation of mPTP opening and redox state. This information may be valuable in designing cardioprotective strategies and pharmacological means in ischemic heart pathologies.

## Figures and Tables

**Table 1:** Mitochondrial phosphoprotein changes in hearts pretreated with NOC-18 versus ischemia.

Mitochondrial Membrane-Bound Proteins	UniProt/SwissProt Number	Fold Change NOC-18/Ischemia	*P*-Value
Mitochondrial pyruvate carrier 1 (MPC1)	P63031	1.64	0.003
NADH dehydrogenase ubiquinone flavoprotein 2 (NDUFV2)	P19234	−1.53	0.009
Very long chain specific acyl CoA dehydrogenase mitochondrial (ACADVL)	P45953	1.58	0.013
Mitochondrial 2 oxoglutarate malatecarrier protein (SLC25A11)	P97700	1.66	0.013
ADP/ATP translocase 1 (SLC25A4)	Q05962	1.57	0.022
ATP synthase subunit alpha mitochondrial (ATP5A1)	P15999	1.55	0.028
Trifunctional enzyme subunit beta mitochondrial (HADHB)	Q60587	1.61	0.032
Electron transfer flavoprotein ubiquinone oxidoreductase mitochondrial (ETFD)	Q6UPE1	1.63	0.041
Cytochrome c oxidase subunit 4isoform 1 mitochondrial (COX4I1)	P10888	1.54	0.042
Cytochrome b c1 complex subunit Rieske (UQCRFS1)	P20788	1.54	0.043
**Mitochondrial Matrix Proteins**			
Pyruvate dehydrogenase E1 component subunit alpha somatic form mitochondrial (PDHA1)	P26284	1.55	0.024
Short chain specific acyl CoAdehydrogenase mitochondrial (ACADS)	P15651	1.58	0.027
Long chain specific acyl CoAdehydrogenase mitochondrial (ACADVL)	P15650	1.59	0.027
Isovaleryl CoA dehydrogenase mitochondrial (IVD)	P12007	1.59	0.030
Succinyl CoA ligase ADP GDP forming subunit alpha mitochondrial (SUCLG1)	P13086	1.62	0.030
Lon protease homolog mitochondrial (LONP1)	Q924S5	1.75	0.032
Hexaprenyldihydroxybenzoatemethyltransferase mitochondrial (COQ3)	Q63159	1.67	0.032
Citrate synthase mitochondrial (CS)	Q8VHF5	1.57	0.034
3-ketoacyl CoA thiolase mitochondrial (ACAA2)	P13437	1.57	0.044
Malate dehydrogenase mitochondrial (MDH2)	P04636	1.54	0.049

The identified proteins were categorized based on subcellular location. Fold change expressed as NOC-18 versus Ischemia (*p* ≤ 0.05 of *t*-test statistics presented in ascending order). (−) indicates decreased phosphorylation level after NOC-18 treatment.
